# Apparently benign craniocervical signs in achondroplasia: “neurologic leftovers” identified through a retrospective dataset

**DOI:** 10.1186/s13023-020-01584-5

**Published:** 2020-10-23

**Authors:** Cory J. Smid, Janet M. Legare, Peggy Modaff, Richard M. Pauli

**Affiliations:** 1 Department of Pediatrics, School of Medicine and Public Health, University of Wisconsin, 1500 Highland Avenue, #359, Madison, WI 53705 USA; 2 The Midwest Regional Bone Dysplasia Clinic, School of Medicine and Public Health, University of Wisconsin, Madison, WI USA

**Keywords:** Achondroplasia, Cervical myelopathy, Cervicomedullary decompression, Craniocervical junction, Natural history, Neurologic disease

## Abstract

**Background:**

Achondroplasia is the most common dwarfing disorder. It can result in a variety of sequelae, including neurologic complications, among which high cervical myelopathy is one of particular concern. However, some individuals with achondroplasia appear to have persistent signs by physical examination that, while they might suggest the presence of high cervical myelopathy, remain isolated, non-progressive and apparently benign. To document and quantify these apparently benign craniocervical signs (ABCS) a cohort of 477 individuals with achondroplasia was retrospectively analyzed and information regarding persistent neurologic features suggestive of high cervical myelopathy was recorded in a REDCap database.

**Results:**

Within this cohort, 151 individuals (31.7%) had neurologic examinations that were in some manner concerning. Of these, 46 (30.5% of the subpopulation) required cervicomedullary decompressive surgery. The remaining 105 had concerning signs by examination but no apparent evidence for clinically significant cervical myelopathy. Of those 105 individuals, 88 (83.8%; 18.4% of the entire population) remained neurologically intact throughout their follow-up, and without clinical sequelae.

**Conclusions:**

It appears that many individuals with achondroplasia, if carefully examined, may demonstrate isolated, initially concerning signs suggestive of cervical myelopathy, but in the vast majority these are benign and do not indicate need for aggressive neurosurgical intervention. Further investigations may help to identify ways to differentiate these benign features from the less common but more problematic true myelopathic ones. We postulate that the “neurologic leftovers” may arise from temporally remote, subtle damage to the spinal cord at the craniocervical junction, which damage otherwise does not reach clinical relevance.

## Background

Achondroplasia is characterized by disproportionate growth of the long bones, resulting in the most common form of short-limbed dwarfism [1]. Additionally, bony abnormalities at the foramen magnum may result in compression of the upper cervical-spine and brainstem [2] that may be manifest as sudden unexpected death or cervical myelopathy [3]. Although the risk for sudden death is highest within the first year of life [4, 5], high cervical myelopathy can present at any age [6]. In young children, classic features suggestive of such cervical myelopathy include persistent hypotonia, asymmetric reflexes and/or strength, ankle clonus, or upgoing response to Babinski stimulation, while beyond infancy features may include increased fatigue, decreased walking endurance and a host of other features [3]. At least 8–17% of individuals with achondroplasia will require cervicomedullary decompression surgery to minimize the effects of compression at the craniocervical junction [8–11].

Due to risks of compression at the craniocervical junction, all individuals with achondroplasia must undergo neurologic assessment including neurologic history and neurologic examination, imaging of the craniocervical junction (by computerized tomography or magnetic resonance imaging), and polysomnography shortly after initial diagnosis [3]. Distinguishing those with need for urgent surgical intervention from infants who display diagnosis-typical motor delays and axial hypotonia remains challenging. Criteria used in our clinics to determine if craniocervical decompression is warranted have been published previously [3, 7].

An abnormal neurologic examination raises concern. However, we began to recognize individuals with persistent but isolated neurologic findings by examination in whom there was no subsequent evidence for development of cervical myelopathy or further evidence for cervicomedullary compression; we refer to these apparently isolated findings as “apparently benign craniocervical signs” (ABCS) or “neurologic leftovers”. Isolated “neurologic leftovers” without further evidence for development of myelopathy have been reported previously only rarely in the literature concerning achondroplasia [12, 13]. However, it was our clinical impression that ABCS are common, but often without consequence on long-term outcome.

Therefore, we conducted a retrospective study of 477 individuals with achondroplasia to assess the frequency, nature, and outcomes of patients with these “neurologic leftovers”.

## Results

### Demographics

**Fig. 1:**
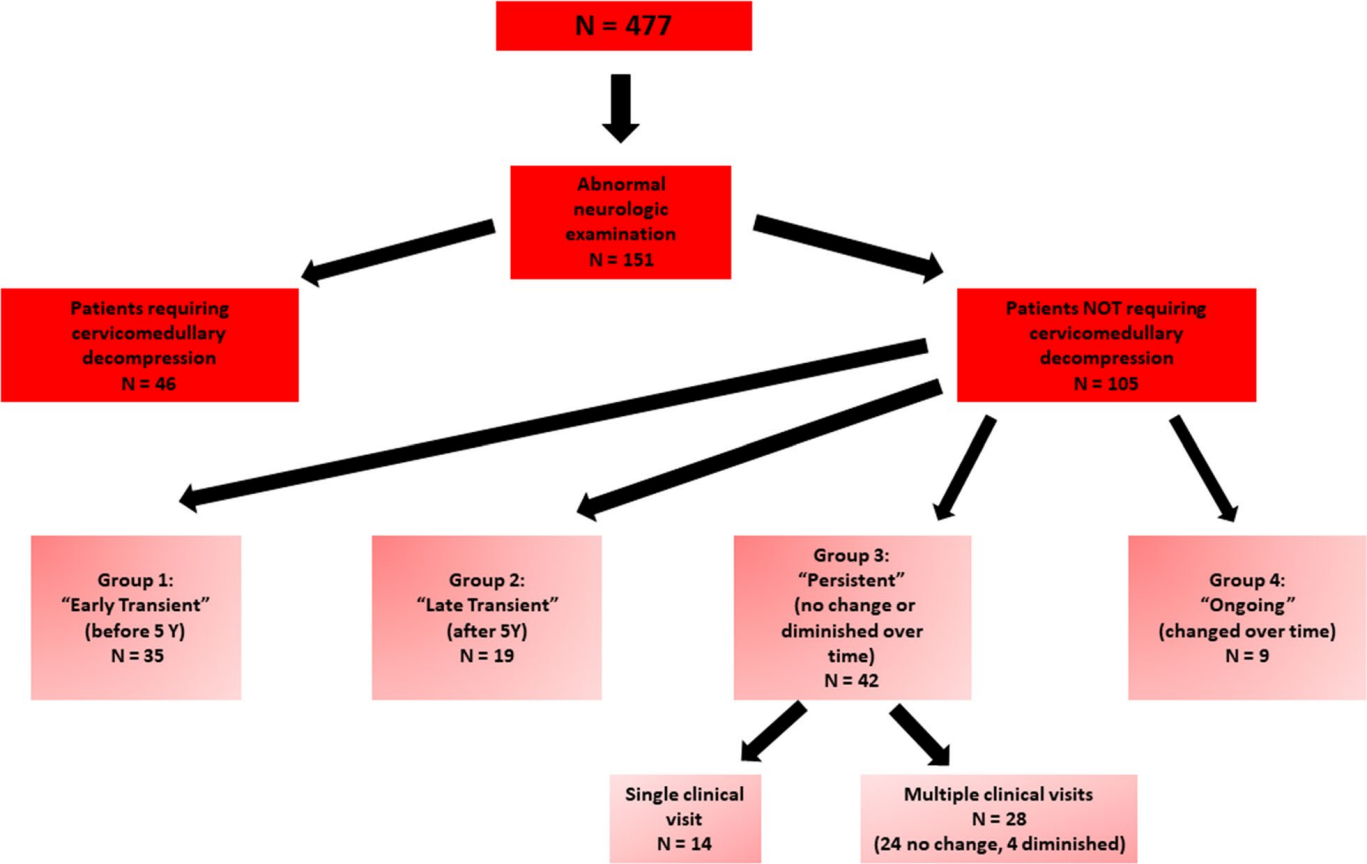
Compartmentalization of the cohort into four groups as defined in the text

Overall, 151 of 477 individuals (31.7%) had initially concerning neurologic examinations. Of these, 46 (30.5% of those with concerning examinations) were excluded from further consideration based on their need for cervicomedullary decompression surgery (Fig. 1). Note that those who were excluded because they underwent decompressive surgery does not include all patients within the entire population who underwent such surgery, since in some instances there was not an antecedent abnormal neurologic examination (e.g. surgery completed because of progressive syrinx development without abnormal neurologic findings), or there was no documentation of any neurologic evaluation, or, in one instance, decompression occurred subsequent to completion of data analysis for this study.

Thus, the remaining cohort of 105 individuals (22.0%) had abnormal findings by neurologic examination, but had not been judged to be in need of cervicomedullary decompression.

Demographic characteristics of the total cohort and the subpopulation of subjects with ABCS are shown in Table 1. A total of 53 males (50.5%) and 52 (49.5%) females had evidence for “neurologic leftovers” (Table 1). The median age of initial recognition of ABCS was 5.3 years. There is no significant difference by sex (*p* = 0.7424), or race/ethnicity (white/non-white *p* = 0.3041) between the total cohort and the subpopulation with ABCS.

**Table 1:** Demographic characteristics

	Total cohort	Percent (%)	Those showing “leftovers”	Percent (%)
Sex				
Male	236	49.5	53	51
Female	241	50.5	52	49
Race/ethnicity				
White	415	87.0	94	90
Black	23	4.8	5	5
Hispanic	21	4.4	3	3
American Indian	2	0.4	1	1
Asian	35	7.3	5	5
Other	1	0.2	0	0
Total “white-alone”	398	83.4	92	88
Total “not-white-alone”	79	16.6	13	12

### Frequency and character of ABCS

The most common abnormalities found by neurologic examination included bilateral ankle clonus, bilateral upgoing response to Babinski stimulation, and unilateral upgoing response to Babinski stimulation (Table 2). Subjects may have presented with multiple types of abnormalities, and therefore the number of events shown in Table 2 exceeds the number of subjects.

**Table 2:** Frequency of neurologic signs of potential concern and proportion showing each sign who had normal or abnormal neurologic outcome

Type of concern	Number of subjects N (% n/105)^a^	Normal outcome, N	Abnormal outcome, N
Excessively brisk reflexes	24 (23%)	19 (19/24, 79%)	5 (5/24, 21%)
Asymmetric reflexes, arms	3 (3%)	1 (1/3, 33%)	*2 (2/3, 67%)*
Asymmetric reflexes, legs	15 (14%)	11 (11/15, 73%)	4 (4/15, 27%)
Clonus, unilateral	27 (26%)	20 (20/28, 75%)	7 (7/27, 25%)
Clonus, bilateral	39 (37%)	33 (33/39, 85%)	6 (6/39, 15%)
Upgoing Babinski response, unilateral	35 (33%)	30 (30/35, 86%)	5 (5/35, 14%)
Upgoing Babinski response, bilateral	39 (37%)	30 (30/39, 77%)	9 (9/39, 23%)
Asymmetric strength, arms	3 (3%)	0 (0/3, 0%)	*3 (3/3, 100%)*
Asymmetric strength, legs	6 (6%)	3 (3/6, 50%)	*3 (3/6, 50%)*

^a^The number of events exceeds the total number of subjects since one patient could display more than one sign

### Change in ABCS over time

A large majority of subjects (91/105, 86.7%) was seen more than once, allowing for tracking of neurologic features over time. Age at which concerns were first identified varied from 2 to 835 m with a mean of 126.0 m (and a median of 60 m). As summarized in Fig. 1, 35 of the 105 individuals (33.3%) had ABCS considered to be *Early Transient*—the abnormal neurologic feature was first recognized prior to 5 years of age but completely disappeared subsequently. Nineteen individuals (18.1%) were considered to have *Late Transient* “leftovers”—the abnormal neurologic finding was initially recognized at or after 5 years of age and completely disappeared over time. Combining the Early Transient and Late Transient groups, the interval between recognition and disappearance of “leftovers” averaged 26.8 m (median 18 m).

A total of 42 individuals (40.0%) were classified as having *Persistent* ABCS—the initial abnormality on neurologic examination remained the same or diminished but did not disappear over time. However, note that within this *Persistent* “leftovers” group, 14 individuals were seen only once, and so, in fact, we cannot determine whether features did or did not subsequently remit in that sub-group. Nine individuals (8.6%) were considered to have *Ongoing* “leftovers”—in these the neurologic examination was always abnormal, and over time the concerns became more prominent or additional features developed. These latter groups, of persistent and ongoing abnormality by examination are those in which concern is likely to be the greatest. For those in the “Persistent” and “Ongoing” groups for whom there were assessments following recognition of the abnormal clinical features, length of follow-up ranged from 1 to 399 m, with a mean of 95.4 m and median of 72 m.

### Outcomes

The outcomes of all subjects with ABCS are summarized in Table 3. Overall, 88 of the 105 (83.8%) subjects with such neurologic findings seemed to be otherwise clinically neurologically intact and had a benign outcome (Table 3). Of the 17 individuals who had abnormal outcomes, the most common abnormalities were partial bladder incontinence (n = 8), partial bowel incontinence (n = 6), and limitation of walking (n = 7), (with the number of total events greater than the total number of subjects because an individual may have had more than one sequela).

**Table 3:** Outcomes by category as defined in the text

Outcomes^a^	Overall number of subjects N (%, N/105)	“Early transient” number of subjects N (%, N/35)	“Late transient” number of subjects N (%, N/19)	“Persistent” number of subjects N (%, N/42)	“Ongoing” number of subjects N (%, N/9)
Normal function	89 (85%)	32 (91%)	17 (89%)	34 (81%)	5 (55%)
Persistent hypotonia	2 (2%)	1 (3%)	0	0	1 (11%)
Persistent disproportionate motor delay	3 (3%)	2 (6%)	0	0	1 (11%)
Asymmetric strength	2 (2%)	0	1 (5%)	0	1 (11%)
Hemiplegia	2 (2%)	0	0	1 (2%)	1 (11%)
Marked limitation of walking	7 (7%)	0	0	4 (10%)	3 (33%)
No independent walking	1 (1%)	0	0	1 (2%)	0
Partial bladder incontinence	8 (8%)	0	1 (5%)	5 (12%)	2 (22%)
Complete bladder incontinence	0	0	0	0	0
Partial bowel incontinence	6 (6%)	0	1 (5%)	3 (7%)	2 (22%)
Complete bowel incontinence	1 (1%)	0	0	0	1 (11%)
Other abnormal	0	0	0	0	1 (11%)

^a^The number of events exceeds the total number of subjects since one patient could be categorized as having more than one

Among the groups categorized as above, not surprisingly the *Ongoing* group was least likely to have a benign neurologic outcome (5/9, 55% normal). These are individuals in whom abnormal neurologic features became more prominent, or additional features appeared over time. Compared with the remaining three groups combined, the *Ongoing* group was less likely to have a normal outcome (*p* = 0.0351). There were no clear statistical differences for the proportion of neurologically intact outcomes among any of the remaining groups. Even in patients in whom one might have the greatest concern [groups *Ongoing* and *Persistent* having been seen more than once (together n = 37)], the outcome was completely benign regarding issues at the craniocervical junction in the large majority (28/37, 75.7%).

In fact, of the 17 individuals subsequently defined as not neurologically normal, only three had craniocervical junction concerns (alone) explanatory of their neurologic problems, one had both craniocervical junction issues and lumbosacral spinal stenosis, and one had craniocervical concerns plus an unrelated concurrent condition (congenital cerebellar hypoplasia). Most of those subsequently defined as being neurologically abnormal developed isolated features of either spinal claudication or lumbosacral spinal stenosis (12/17, 71%).

## Discussion

Persistent but apparently benign signs of possible cervical myelopathy appear to be quite common in those with achondroplasia. If careful neurologic examinations are undertaken, 22% of all individuals with achondroplasia (excluding those with clearly actionable craniocervical junction compression in infancy) show evidence for what one might think are worrisome features referable to the upper cervical spinal cord. Yet in the vast majority, no progression arises and no clinically relevant or disabling characteristics develop. That is, most often these neurologic ABCS can be considered benign findings.

Previous descriptions similar to what we report here are rare. Reid et al. [12] may have been the first to recognize that neurologic examination is not completely specific in assessment of possible cervicomedullary compression. More recently, this phenomenon may have been described in one instance by Sanders et al. [13]. In that retrospective study of 49 patients with achondroplasia seen between 1997 and 2017, one patient had a neurologic examination of concern but without evidence for cord compression by magnetic resonance imaging assessment [13]. Why more instances have not been noted in the literature is unclear. Pro forma examinations will fail to identify subtle neurologic abnormalities as were present in many of the individuals described here. Small numbers of patients seen within a particular clinic may mean uncertainty about what is and what is not within normal limits for a child with achondroplasia.

### The craniocervical junction in achondroplasia

Individuals with achondroplasia are at an increased risk of neurological sequelae related to compression at the craniocervical junction [4, 5]. The foramen magnum is frequently small and “key-hole” shaped, constricting and compressing the upper cervical spine and brainstem. Within the first 4 years of life, children with achondroplasia are at an increased risk for sudden death secondary to acute or chronic compression of the upper cervical spine and brainstem [5] with the greatest risk arising in the first year of life [4], while complications due to cervical myelopathy, however, may present at any age [6]. Individuals presenting with unequivocal risk factors for clear-cut cervical myelopathy [6] or sudden infant death [7] need suboccipital decompression surgery.

### When should apparently isolated neurologic signs be cause for concern?

While there are some individuals who present with unequivocal progression of features of high cervical myelopathy in whom urgent neurosurgical intervention is appropriate, discovery of isolated, non-progressive and apparently benign neurologic features is considerably more common. Of those, 46 (30.5% of those with abnormal neurologic examinations) showed other features (by imaging, of polysomnography, onset of acute life-threatening events) that demonstrated that cervicomedullary decompression surgery was needed. The remaining 105 of 151 (69.5%) had neurologic examinations which initially were concerning but did not develop further characteristics warranting neurosurgical intervention. In fact, in many (55/105, 52.4%) the unequivocally abnormal signs were transient. In many others (33/105, 31.4%) they persisted but without progression. In only a small minority (17/105, 16.2%) were any significant neurological concerns eventually present. Even in those, although categorized as having progressive issues, most had subsequent features *unrelated* to the upper cervical spine. Indeed, the vast majority of individuals with ABCS (83.8%) had a normal neurologic outcome. Individuals with initial presentations including asymmetric reflexes or asymmetric strength of the arms or legs appear to be the least likely to have a normal outcome (Table 2). Other predictors of a poor outcome include developing new features, or features becoming more prominent over time (Table 3).

### Why might such “leftovers” arise?

At this time there is no clear etiologic explanation for these ABCS. One attractive hypothesis is that these signs may reflect subtle, temporally remote damage to the upper cervical spinal cord [3]. Repetitive, minor compression from uncontrolled head movement, in combination with infantile macrocephaly, marked hypotonia, and poor head control may more often result in subtle injury than has been previously recognized.

It is also uncertain how these clinical examination features relate to the imaging features described by Brouwer et al. [14] and van Dijk et al. [15]. This group demonstrated a remarkable frequency of cervical high-intensity intramedullary (CHII) lesions by magnetic resonance imaging (MRI) in adults with achondroplasia. This group initially examined MRIs of 25 adults with achondroplasia and suspected neurologic lumbar spinal claudication. CHII lesions located between C1 and C2 were noted in 64% (16/25), without evidence for cervical spinal cord compression [15]. Since this initial cohort was ascertained because of neurologic concerns (although not of the craniocervical junction), subsequently Brouwer et al. [14] analyzed the MRIs of 18 subjects with achondroplasia who explicitly showed no clinical signs of cervical myelopathy. In this population, too, CHII lesions were common, observed in 39% (7/18), with only one of these individuals having anatomic evidence for compression at the craniocervical junction. We think it is conceivable that both the subtle clinical features described here and the imaging findings described in [14, 15] may have a common origin.

### Limitations

This study was limited to individuals with neurologic examinations of concern, and who ultimately were judged not to require cervicomedullary decompression. Indications for cervicomedullary decompression outside of neurologic examinations of concern, such as abnormalities by polysomnography or MRI findings were not addressed. Additionally, patients presenting with neurologic examinations of concern only *after* cervicomedullary decompression are not be included in this study.

It is conceivable that certain patients with poor neurologic outcomes may have benefited from cervicomedullary decompression, yet surgery was never completed due to limited surveillance of the craniocervical junction. These patients were included in this study.

### Recommendations

In order to minimize the risks associated with compression at the craniocervical junction, all individuals with achondroplasia must undergo a neurologic assessment including: neurologic history and neurologic examination, imaging of the craniocervical junction, and polysomnography in early infancy [3]. However, if *isolated* abnormalities are noted by neurologic examination at this point or subsequently, without other evidence for cervical compression, then periodic clinical reassessment may be all that is warranted, with special attention paid to the type of abnormality, and the changes of neurologic features over time. If these remain *isolated* and *non-progressive,* they should not precipitate a rush to neurosurgical intervention. Those with asymmetries, those who develop new features, or whose features become more prominent over time should be more closely monitored, with additional neuroimaging to ensure that timely intervention, when necessary, is initiated.

## Conclusions

Isolated “neurologic leftovers” or ABCS are often a common, benign finding in achondroplasia. Many individuals may display such ABCS when carefully evaluated yet only a very small subset appear to have clinically relevant neurologic sequelae related to high cervical myelopathy. These ABCS may reflect subtle, remote damage to the upper cervical spinal cord due to repetitive, minor compression from uncontrolled head movement in infancy.

The vast majority of individuals with these “neurologic leftovers” will have a normal outcome. Individuals with (1) asymmetric reflexes or asymmetric strength of the arms or legs or (2) development of new features or (3) features which become more prominent over time, appear to be less likely to have a normal outcome, and should be monitored closely.

## Methods

This study was reviewed and approved by the Institutional Review Board of the University of Wisconsin-Madison School of Medicine and Public Health, Madison, WI.

All individuals with a diagnosis of achondroplasia who were seen in the Midwest Regional Bone Dysplasia Clinic between 1980 and 2017 were retrospectively analyzed. All individuals enrolled in this study had confirmed molecular, radiographic, and/or clinical diagnoses of achondroplasia. All were comprehensively clinically assessed by a physician experienced in distinguishing achondroplasia from other skeletal dysplasias. Individuals were excluded if they had confirmed molecular, radiographic, and/or clinical diagnoses of homozygous achondroplasia, compound heterozygosity (i.e. achondroplasia + hypochondroplasia) or double heterozygosity (achondroplasia + another bone dysplasia [16, 17].

Data were collected from review of all available medical records and entered into a supplement to a REDCap database [18] developed for a multi-center natural history study of achondroplasia [19]. Data regarding the presence, characteristics, and changes of neurologic examinations of concern were entered, as were data related to overall outcome.

Neurologic examinations were performed at each clinical visit. Concerning neurologic findings were defined as the following: excessively brisk reflexes; asymmetric reflexes in arms; asymmetric reflexes in legs; unilateral ankle clonus; bilateral ankle clonus; unilateral upgoing response to Babinski stimulation; bilateral upgoing response to Babinski stimulation; asymmetric strength in arms; and asymmetric strength in legs. In addition, note was made of hypotonia outside of the range expected for individuals with achondroplasia, and for disproportionate motor delays. Prior to 6 months of age, neurologic examinations were only considered abnormal if asymmetries were present since other neurologic abnormalities such as ankle clonus may persist for the first several months of life, but then disappear spontaneously around 6 months of age [20]. If patients were seen multiple times, changes in neurological findings could be documented over time. Individuals were grouped in categories based on the change in neurological findings over time.

This study excludes all individuals without neurologic examinations of concern. In addition, individuals who underwent cervicomedullary decompression are excluded (Fig. 1), since those requiring cervicomedullary decompression had evidence for risk of sudden death, progressive cervical myelopathy and/or additional concerning features (e.g. abnormal imaging), and so did not demonstrate isolated, presumably benign ABCS.

## Data Availability

HIPAA requirements preclude sharing of primary data upon which this study is based.

## Abbreviations

ABCS: Apparently benign craniocervical signs
CHII: Cervical high intensity intramedullary (lesions)
MRI: Magnetic resonance imaging
REDCap: Research electronic data capture
